# Mapping neural dynamics underlying saccade preparation and execution and their relation to reaction time and direction errors

**DOI:** 10.1002/hbm.24922

**Published:** 2020-01-09

**Authors:** Sonya Bells, Silvia L. Isabella, Donald C. Brien, Brian C. Coe, Douglas P. Munoz, Donald J. Mabbott, Douglas O. Cheyne

**Affiliations:** ^1^ Program in Neurosciences and Mental Health, The Hospital for Sick Children Research Institute Toronto Ontario Canada; ^2^ Institute of Medical Sciences, Institute of Biomaterials and Biomedical Engineering, University of Toronto Toronto Ontario Canada; ^3^ Centre for Neuroscience Studies Queen's University Kingston Ontario Canada; ^4^ Department of Psychology University of Toronto Toronto Ontario Canada; ^5^ Department of Medical Imaging University of Toronto Toronto Ontario Canada

**Keywords:** antisaccade, frontal cortex, inhibition, magnetoencephalography, parietal cortex

## Abstract

Our ability to control and inhibit automatic behaviors is crucial for negotiating complex environments, all of which require rapid communication between sensory, motor, and cognitive networks. Here, we measured neuromagnetic brain activity to investigate the neural timing of cortical areas needed for inhibitory control, while 14 healthy young adults performed an interleaved prosaccade (look at a peripheral visual stimulus) and antisaccade (look away from stimulus) task. Analysis of how neural activity relates to saccade reaction time (SRT) and occurrence of direction errors (look at stimulus on antisaccade trials) provides insight into inhibitory control. Neuromagnetic source activity was used to extract stimulus‐aligned and saccade‐aligned activity to examine temporal differences between prosaccade and antisaccade trials in brain regions associated with saccade control. For stimulus‐aligned antisaccade trials, a longer SRT was associated with delayed onset of neural activity within the ipsilateral parietal eye field (PEF) and bilateral frontal eye field (FEF). Saccade‐aligned activity demonstrated peak activation 10ms before saccade‐onset within the contralateral PEF for prosaccade trials and within the bilateral FEF for antisaccade trials. In addition, failure to inhibit prosaccades on anti‐saccade trials was associated with increased activity prior to saccade onset within the FEF contralateral to the peripheral stimulus. This work on dynamic activity adds to our knowledge that direction errors were due, at least in part, to a failure to inhibit automatic prosaccades. These findings provide novel evidence in humans regarding the temporal dynamics within oculomotor areas needed for saccade programming and the role frontal brain regions have on top‐down inhibitory control.

## 1 INTRODUCTION

The antisaccade task has proven to be an important tool to measure inhibitory control because it requires participants to inhibit an automatic, visually guided saccade toward a suddenly appearing visual stimulus (prosaccade), and instead generate a voluntary antisaccade in the opposite direction (Hallett, 1978; Hallett & Adams, 1980). Reaction times within antisaccades are longer than prosaccades (Hallett, 1978) and this delay is linked to top‐down inhibition of a prosaccade on antisaccade trials (Everling, Dorris, & Munoz, 1998; Everling & Munoz, 2000; Guitton, Buchtel, & Douglas, 1985; Zhang & Barash, 2000). Monkey neurophysiological and human functional neuroimaging studies have implicated several cortical and subcortical areas, including the basal ganglia, thalamus, superior colliculus (SCs), and cerebellum, which are involved in saccadic eye movements (Hikosaka, Nakamura, & Nakahara, 2006; Munoz & Everling, 2004; Schall, 2004; Watanabe & Munoz, 2011). Briefly, once the stimulus appears, a visual signal is sent to the primary visual cortex and the SCs. Visual information is then processed in extra‐striate visual structures, which connects to motor structures and the parietal eye field (PEF; lateral intraparietal area [LIP] in monkeys), to affect movement for prosaccades. The PEF projects to subcortical areas, SCs and caudate nucleus (Watanabe & Munoz, 2010) and frontal cortical oculomotor areas, such as frontal eye fields (FEF) (Everling & Munoz, 2000), supplementary eye fields (SEF) (Schlag‐Rey, Amador, Sanchez, & Schlag, 1997) and the dorsolateral prefrontal cortex (DLPFC) (Johnston & Everling, 2006a). For a more comprehensive description on this network, see (Coe, Trappenberg, & Munoz, 2019; Coe & Munoz, 2017). Prior functional magnetic resonance imaging (fMRI) studies have identified key cortical areas—the PEF and FEF—involved in antisaccade and prosaccade generation and inhibition (Alahyane, Brien, Coe, Stroman, & Munoz, 2014; Connolly, Goodale, Menon, & Munoz, 2002; Fernandez‐Ruiz et al., 2018; Luna et al., 2001; Munoz & Everling, 2004). Inhibition and generation of a saccade in the opposite direction of a visual stimulus involve suppression of these key areas contralateral to the stimulus followed by visual remapping of the stimulus properties to the ipsilateral side of the brain (i.e., vector inversion) (Brown, Goltz, Vilis, Ford, & Everling, 2006; Chikazoe, Konishi, Asari, Jimura, & Miyashita, 2007; Ettinger et al., 2008; Munoz & Everling, 2004; Watanabe & Munoz, 2011; Wegener, Johnston, & Everling, 2008). Monkey neurophysiological studies have characterized dynamics for neurons in specific regions of the frontal and parietal cortex (Everling & Munoz, 2000; Johnston & Everling, 2006b; Schlag‐Rey et al., 1997; Zhang & Barash, 2000), although this work was typically limited to recording single neurons within individual monkeys. Noninvasive measurements of brain activity related to saccade control in humans using fMRI are hampered by the poor temporal resolution of the hemodynamic signal compared to neuronal recordings and the fact that millions of neurons are represented within a typical voxel. The limitations of fMRI restrict the ability to look at individual neural dynamics of the inhibitory top‐down control as manifested through the saccade control network.

Despite many previous studies demonstrating the importance of the FEF and PEF for saccade preparation in monkeys (Everling & Munoz, 2000; Johnston & Everling, 2006b; Schlag‐Rey et al., 1997; Zhang & Barash, 2000), the neural timing of these areas in humans is still fairly unknown. Evaluating temporal differences between correct prosaccades and antisaccades in healthy humans can be used to classify signals of response inhibition and saccade generation. Further applications include developing a classification scheme for neural degeneration. The primary goal in this study is to utilize the high temporal resolution of magnetoencephalography (MEG) to characterize and distinguish the relative timing of neural activity needed for inhibitory control within the PEF and FEF in the ipsilateral and contralateral hemispheres, while healthy young adults perform an interleaved prosaccade and antisaccade task. This task provides measures of saccade reaction time (SRT; the time from stimulus appearance to the first saccade) and direction errors (looking toward the stimulus on an antisaccade trial), which provide insight into cortical areas important for saccade suppression (Munoz & Everling, 2004). Young adults tend to better prosaccade and antisaccade performance compared to healthy children and older adults: they tend to have faster correct anti‐SRTs and generate fewer direction errors (Coe & Munoz, 2017). Visually triggered prosaccades are accompanied by activation of the contralateral PEF (Baizer, Ungerleider, & Desimone, 1991; Sereno, Pitzalis, & Martinez, 2001) and FEF (Bruce & Goldberg, 1985). However, for antisaccade trials, two separate saccade mechanisms are activated, and the first of these to surpass a threshold triggers a saccade. The first mechanism is initiated with the appearance of the stimulus, launching neural activity contralateral to the stimulus that is associated with the generation of an automatic prosaccade, while the other mechanism is launched in the opposite hemisphere, ipsilateral to the stimulus by the inversion of the stimulus vector to initiate a voluntary anti‐saccade. Thus, to perform a correct antisaccade, the mechanism associated with generation of the automatic prosaccade must be inhibited to allow the neural activity associated with the generation of the voluntary antisaccade response to surpass the threshold. However, if inhibition is unsuccessful, an automatic prosaccade (direction error) will be triggered toward the stimulus. The processes underlying the suppression of automatic prosaccades can be better understood through investigation of the frequency and timing of these direction errors, granting greater insight into the mechanisms of inhibitory control.

Two areas that might have a crucial role in vector inversion are the PEF and FEF. The PEF is the interface between sensory and motor processing (Colby, Duhamel, & Goldberg, 1996; Gnadt & Andersen, 1988). Previous research with monkeys found that neurons within the LIP, the monkey homolog to the PEF, represented stimulus location; with few neurons representing the direction of movement (Gottlieb & Goldberg, 1999). Conversely, Zhang and Barash (2000, 2004) demonstrated that PEF neurons responded to both location and direction, whereby the PEF saccade neurons when aligned to the response field activated 50ms after the visual neurons on the contralateral side of the brain (contralateral to the visual stimulus). Human electrophysiological (Everling, Spantekow, Krappmann, & Flohr, 1998) and fMRI (Furlan, Smith, & Walker, 2016) data have demonstrated a switch from contralateral PEF to ipsilateral PEF activity on antisaccade trials demonstrating how PEF is involved in response preparation and motor planning. The FEF is also important for antisaccade generation and is involved in vector inversion: Sato and Schall (2003) found different neurons within the FEF to be involved in visual selection and saccade selection.

Although these studies show that PEF and FEF are involved in vector inversion, they did not examine both regions simultaneously to compare the relative activation latencies that underlie these hemispheric switches needed for generation of correct antisaccades. An MEG study by Moon et al. (2007) attempted to show this but failed to demonstrate vector inversion in the right hemisphere. This unexpected result of only detecting vector inversion in the left hemisphere could be due to a number of reasons: too low a cortical mesh density could have effected the final forward model during MEG processing (Henson, Mattout, Phillips, & Friston, 2009) or as the authors suggested, using regions of interest (ROIs) reduced the ability to detect directionally selective activity in the right hemisphere, which is most likely due to a low signal‐to‐noise ratio. Here, we build on previous electrophysiological work and provide the first evidence in humans for the theorized vector inversion model, which occurs within the PEF and FEF in both hemispheres.

It has been proposed that variability within SRT is related to variability in the time course of activation within the oculomotor network (Coe & Munoz, 2017). If SRT variability is related to fluctuations in neural activity related to vector inversion, then there should be a relationship between SRT and neural processing within the parietal and frontal regions after the stimulus presentation. In the current study, we evaluated the contribution of neural variability to SRT. To meet this objective, we separated trials into fast and slow antisaccades based upon a median split of SRT within each participant. We hypothesized that neural timing within the FEF and PEF would differ between fast and slow SRTs after stimulus presentation but before saccade initiation. Grouping SRT into fast and slow bins also allowed us to directly relate premovement MEG activity and saccade preparation by correlating its activity with SRT. Previous work has shown both a positive relationship in human MEG (Sestieri, 2008) and a negative relationship in human EEG and monkey neurophysiological studies (Everling & Munoz, 2000; Hanes & Schall, 1996; Papadopoulou, Evdokimidis, Tsoukas, Mantas, & Smyrnis, 2010; Schall, 2015) between the FEF activity and SRT. The variability in saccade behavior is proposed to be reliant on top‐down behavior such as decision‐making, pre‐motor, and response inhibition (Hanes & Schall, 1996; Thompson, Hanes, Bichot, & Schall, 1996). If MEG activity within the FEF reflects the extent to which one is reliant on top‐down processes for saccadic movement, then we should observe less neural activity for fast SRTs and more activity for slower SRTs. The ability to measure response inhibition is crucial to understanding flexible, adaptive and goal‐directed behavior.

Although previous studies demonstrate the importance of the FEF and PEF for saccade preparation (Everling & Munoz, 2000; Johnston & Everling, 2006b; Schlag‐Rey et al., 1997; Zhang & Barash, 2000), the timing by which these areas come online during prosaccade and antisaccade execution in humans is still fairly unknown. Many studies have looked at prosaccadic activity only and found increased posterior activity within: (a) EEG electrodes (Balaban & Weinstein, 1985; Csibra, Johnson, & Tucker, 1997; Kurtzberg & Vaughan, 1982; Tzelepi, Lutz, & Kapoula, 2004; Tzelepi, Laskaris, Amditis, & Kapoula, 2010; Weinstein, Balaban, & Verhoeve, 1991), (b) MEG dipole moments (Natsukawa & Kobayashi, 2012), or (c) MEG sources (Sestieri, 2008). A number of EEG studies have focused on differences between prosaccade and antisaccade trials and have observed that prior to movement, antisaccade trials had greater posterior contralateral electrode activity (i.e., PEF) (Everling, Spantekow, et al., 1998; Papadopoulou et al., 2010; Richards, 2013) and frontal electrode activity contralateral to movement (McDowell et al., 2005; McDowell, Dyckman, Austin, & Clementz, 2008; Richards, 2003). This same pattern was seen in a few MEG studies (Herdman & Ryan, 2007; Moon et al., 2007), where greater PEF activity in prosaccade trials and greater FEF activity in antitrials were found. Another study demonstrated increased FEF activity poststimulus display (McDowell et al., 2005). Although these studies provide important information regarding spatial–temporal differences between prosaccade and antisaccade behavior premovement, they do not affirm the specific timing when frontal and parietal regions come online with respect to one another to support correct saccadic behavior. An additional goal in the current study was to identify the time course of activation within the PEF and FEF for prosaccade and antisaccade execution, which are time‐aligned to saccade onset. Based on evidence from fMRI (Connolly et al., 2002; DeSouza, Menon, & Everling, 2003; Furlan et al., 2016) and MEG (Herdman & Ryan, 2007) studies, we predicted that prosaccade trials would have more pronounced PEF involvement and antisaccade trials would have more pronounced ipsilateral and contralateral FEF involvement than prosaccade trials.

A link between antisaccade behavior and neural activity has been assessed in monkey FEF, LIP, and SCs; however, linking this underlying neural circuit to the MEG signals remains a challenge. Neurons within contralateral LIP, FEF, and SCs must be inhibited (Everling & Munoz, 2000) and ipsilateral FEF and SCs neurons must be activated (Everling, Dorris, Klein, & Munoz, 1999; Everling & Munoz, 2000) in order to drive voluntary antisaccade behavior. Furthermore, if there is insufficient inhibition of saccade neurons in the contralateral FEF, direction errors occur. By exploring differences in premovement responses between correct prosaccade and antisaccade trials processes needed for inhibition can be revealed. Using MEG, Herdman and Ryan (2007) demonstrated the importance of FEF in correct antisaccade movements; however, they were unable to untangle the differences between ipsilateral and contralateral FEF activity. In this study, we expand on these results by exploring the differences between prosaccade and antisaccade trials in addition to stimulus side (left or right) to reveal the mechanisms needed for inhibition. Furthermore, it is still unknown how premovement activity within human ipsilateral and contralateral FEF in correct antisaccade trials is related to direction errors. Given the relationship between FEF activity and antisaccade preparation (Connolly et al., 2002; DeSouza et al., 2003; Furlan et al., 2016), we hypothesized that greater neural activity within the contralateral FEF before an antisaccade movement will covary with the proportion of direction errors made in anti‐saccade trials. Concomitantly, a decrease in activity will represent how well a participant can consistently inhibit an automatic saccade, thus making fewer direction errors. Assessing the temporal dynamics and activity of the frontoparietal circuit will expand our understanding of the processes needed for inhibitory control.

## 2 MATERIALS AND METHODS

### 2.1 Participants

Fourteen healthy right‐handed adults participated in this study (age 26.8 ± 2.6 years; 7 females; Edinburgh handedness inventory = 87.5 ± 14.7) (Oldfield, 1971; Ransil, 1994; White & Ashton, 1976). All participants had normal vision and provided informed consent using protocols approved by the Hospital for Sick Children Research Ethics Board.

### 2.2 Interleaved prosaccade and antisaccade task

**Figure 1:**
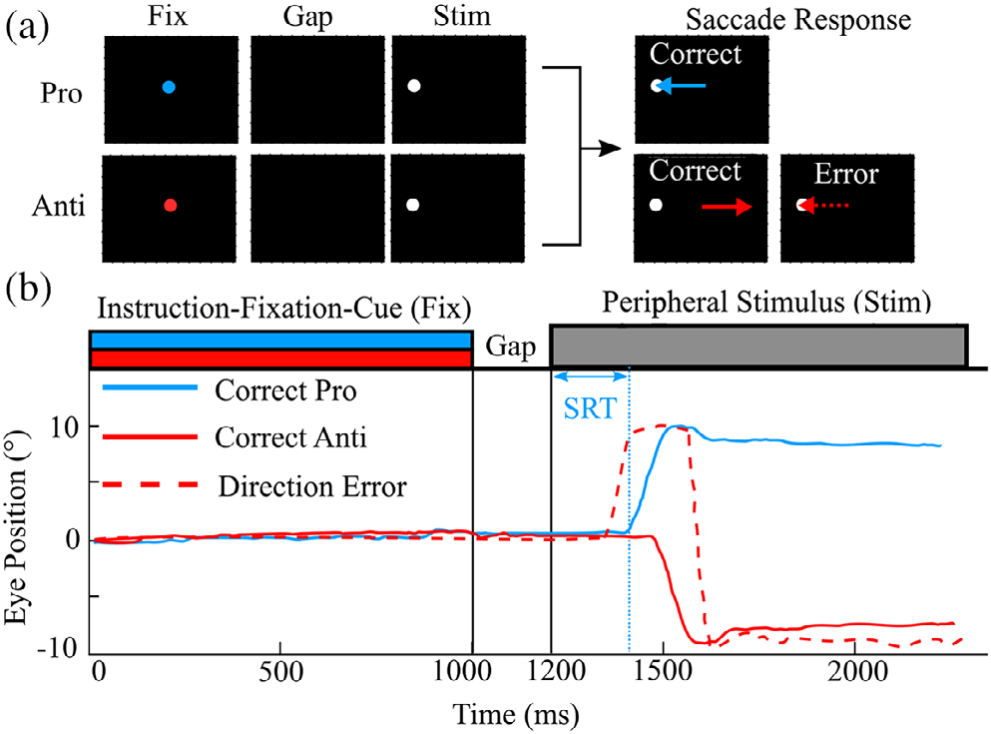
Pro/antisaccade task. (a) Representation of stimuli for two of the four trial types. A central instructional‐fixation cue (Fix) was presented for 1,000ms. A green Fix instructed a pro trial (blue) and a red instructed an antitrial. This was followed by a gap period (black screen) of 200ms and then a white peripheral stimulus (Stim) 10° either left or right of central for 1,000ms (stimulus on the right is not shown). The blue arrow indicates a correct prosaccade and the solid red arrow indicates a correct anti saccade, while the dashed red arrow indicates a direction error. (b) Representation of task timing and sample eye traces depicting a correct prosaccade trials (solid blue), a correct antisaccade (solid red), and a direction error (dashed red). Saccade reaction time (SRT) is the time between when the Stim was displayed and the start of a saccadic movement

Participants performed three blocks of randomly interleaved prosaccade and antisaccade trials (180 trials/block, each block lasting approximately 7min) (Figure 1a). Each trial started with a circular fixation‐instructional cue (Fix) presented in the center of the screen for 1,000ms. A green fixation cue instructed a prosaccade trial and a red fixation cue instructed an antisaccade trial. The fixation‐instructional cue disappeared and a 200ms gap with no stimuli occurred before the target stimulus (white circle) appeared either to the right or to left side at 10° eccentricity. A gap period has been shown to increase the probability of participants generating more “automatic” saccades, shorten SRTs, increase the number of antisaccade direction errors, and increase the number of express prosaccades (Fischer & Weber, 1997; Munoz, Broughton, & Goldring, 1998; Munoz & Corneil, 1995) (see example in Figure 1b). Participants had 1,400ms before the start of the next trial to execute a saccade and re‐establish central fixation on the fixation‐instructional cue.

### 2.3 MEG recordings

Neuromagnetic activity was recorded using a 151‐channel CTF MEG system (600 samples/s, DC‐200Hz; MISL, Coquitlam, BC, Canada) in a magnetically shielded room while simultaneously recording high‐resolution eye tracking (MEG compatible 500Hz Eyelink 1,000; SR Research Ltd, Oakville, ON, Canada). Continuous MEG data were collected while participants sat upright in an adjustable chair, while stimuli were displayed on a screen using a LCD projector on a back‐projection (refresh rate 60Hz). Visual stimuli, eye positions, and trial markers (e.g., beginning of the trial) were exported and synchronized with MEG collection. Eye position was calibrated using a nine‐point array that covered most of the visual field. Small coils placed at fiducial locations (nasion and pre‐auricular points) were used to monitor head position during recording. Fiducial locations were used to co‐register source images to the participants' magnetic resonance imaging (MRI). Structural T1‐weighted MR images were obtained for each participant using a Siemens 3T Prisma scanner.

### 2.4 Eye‐tracking behavioral analysis

Prosaccade and antisaccade trials were analyzed using custom written scripts in MATLAB (MathWorks), which evaluated SRT, express saccades, direction errors, and intra‐participant variability. The fixation period prior to stimulus presentation was evaluated to determine whether participants held their gaze on the fixation point defined as less than 2° from the fixation point. Microsaccades were defined as saccades less than 2° from the fixation point and were excluded in saccade detection. The onset and termination of a saccade were defined as when the eye velocity exceeded 30°/s. Saccades made prior to 90ms were classified as anticipatory and were not included in any analyses because they were equally likely to be made in either direction (Munoz & Istvan, 1998). Correct prosaccades were defined as saccades executed toward the stimulus and landing within 2° from the stimulus, while correct antisaccades were executed away from the stimulus and landing within 2° of the opposite location of the stimulus. Express latency saccades, which are the shortest visually triggered saccades (Fischer et al., 1993; Munoz et al., 1998), were defined to have SRTs between 90 and 140ms. Regular latency saccades were defined as any correct SRT greater than 140ms and less than 800ms. Direction errors were defined as regular latency saccades executed away from the stimulus during prosaccade trials and landing within 2° of the opposite location of the stimulus or toward the stimulus and landing within 2° of the stimulus during antisaccade trials. Direction error (DE) was calculated by dividing the total number of direction errors by the total number of trials. Intra‐participant variability for SRT (cvSRT) was calculated using the coefficient of variation for correct trials (SD/mean × 100).

To compare prosaccade and antisaccade task measures, log‐transformed SRT and cvSRT were compared using a 2 by 2 within‐participant ANOVA for between‐ and within‐task comparisons (factors were task and stimulus side, respectively) using *R* (R Core Team, 2018). Both percent direction errors and percent express saccades violated the normality assumption required for traditional regression or ANOVA and were also zero‐inflated as some participants did not have any direction errors or express saccades. Thus, a negative binomial regression within R with a log link function (*glmer.nb*) was used to analyze the number of direction errors or express saccades. The main effects were task and side of the visual stimulus and random effects were intercepts for participants in the direction error model. A similar model was used for express saccades, with the exception of task since express saccades occurred for prosaccade trials only.

The following exclusion criteria (i.e., invalid trials) were used for both prosaccade and antisaccade trials across all three blocks for MEG source localization: (a) failure to fixate during the fixation‐instructional cue period (when eye movements are greater than 2° from fixation point); (b) execution of a slow saccade, defined as greater than 800ms after stimulus appearance; (c) execution of multiple saccades (>3) during the response period, such as in an antisaccade trial when a saccade is made toward the stimulus followed by a correction saccade and again with a saccade back to the stimulus; (d) anticipatory saccades (SRT less than 90ms; Munoz et al., 1998); (e) direction errors for prosaccade and antisaccade trials; and (f) trials in which eye‐tracking was lost (e.g., eye blinks and head movements).

### 2.5 Event‐related MEG analysis

Continuous head localization was used to monitor head motion throughout the recording and trials were rejected if head motion exceeded 5mm or peak‐to‐peak changes were greater than 10 picoTesla. All three blocks were combined prior to source analysis. Localization of neuromagnetic signals was carried out using a scalar event‐related beamforming (ERB) algorithm and single‐sphere head model integrated within the *BrainWave* Matlab toolbox (Jobst, Ferrari, Isabella, & Cheyne, 2018). A scalar minimum‐variance beamformer (Cheyne, Bakhtazad, & Gaetz, 2006; Cheyne, Bostan, Gaetz, & Pang, 2007; Robinson & Vrba, 1999) was used to generate whole‐brain spatiotemporal source images of evoked cortical activity (1–30Hz) time‐locked (aligned) to: (a) both visual fixation‐instructional cue and stimulus onset and (b) saccade movement in order to measure differences in saccade preparation or response. A 4‐s time window centered around each participant's time‐aligned cue or saccade was used to compute the data covariance used for estimating the beamformer spatial filter weights from the single trial data. Each trial was visually inspected for significant oculomotor artifact and removed if found. It should be noted that MEG beamforming is much less susceptible to eye‐movement contamination than EEG (Yuval‐Greenberg, Tomer, Keren, Nelken, & Deouell, 2008) and has been found to separate oculomotor artifacts from cortical sources sufficiently distant from the eyes and more reliably locate saccade muscle noise in the extra‐orbital region (Carl, Açik, König, Engel, & Hipp, 2012) (See Supplementary Figure S1, e.g., sensor data centered around eye‐movement).

Source orientation at each location was based on maximal source power output of the beamformer weights over the covariance time window. The following volumetric source images were created with 4mm resolution and noise‐normalized to units of pseudo‐Z using a noise constant of 3 femtoTelsa/√Hz (Jobst et al., 2018):

1. Saccade‐preparation (stimulus‐aligned): time from 100ms before to 1,500ms following the instructional‐fixation‐cue appearance (this range was used to look at both instructional‐fixation‐cue and stimulus‐aligned responses. The stimulus appeared 1,200ms after instructional‐fixation‐cue appearance, see Figure 1) for eight different response types: fast and slow saccades (median split of SRT distribution) in the following conditions: pro‐left, pro‐right, anti‐left, and anti‐right. Separate sets of images (weights) allowed for optimal localization of ipsilateral and contralateral FEF peaks.
2. Saccade‐execution (saccade‐aligned): time from 500ms before to 500ms after saccade onset every 5ms for the four different response conditions: pro‐left, pro‐right, anti‐left, and anti‐right. Separate sets of images (weights) allowed for optical localization of the ipsilateral and contralateral FEF peaks.

Peaks of cortical activation across space and time within the saccade‐preparation (stimulus‐aligned) and saccade‐execution (saccade‐aligned) time windows were determined and source direction was aligned across participants. Source images were spatially normalized to the Montreal Neurological Institute (MNI) (T1) template brain with Statistical Parametric Mapping (SPM 12: Wellcome Institute of Cognitive Neurology, London, UK; Ashburner, 2009) for group averaging and alignment to the Talairach atlas (http://www.talairach.org) for brain area labeling using the group analysis tool in BrainWave. We focused on known cortical areas that are consistently active across other prosaccade and antisaccade imaging studies and are important for the inhibition of antisaccades (e.g., FEF and PEF). We used coordinates from previous fMRI studies (Alahyane et al., 2014; Brown, Vilis, & Everling, 2007; Fernandez‐Ruiz et al., 2018; Munoz & Everling, 2004) to extract source waveforms. These waveforms were used to determine which time point to carry forward in further analyses. The resulting group ERB images were thresholded (*p* < .05) using a nonparametric permutation test adapted for beamformer source images (Singh, Barnes, & Hillebrand, 2003) and significant sources were inspected. Significant sources in the group‐averaged data were then used to generate virtual sensors (constrained to a search radius of 10 mm) based on each participant's data by inverse transforming from MNI space to individual participant's MEG coordinate system and then averaged to view the entire group time course of activity for these locations. For anatomical visualization, ERB images were interpolated onto high‐resolution cortical mid‐surfaces extracted for each individual participant using CIVET version 1.1.12 (https://portal.cbrain.mcgill.ca) (Kim et al., 2005).

#### 2.5.1 Saccade‐preparation (stimulus‐aligned) analysis

For event‐related oculomotor and sensory fields of source‐reconstructed data for each participant, evoked‐response time courses occurring prior to and after saccade movements were extracted in noise‐normalized (pseudo‐Z) units.

Given our hypothesis that the neural time course of activation for saccade preparation will differ between prosaccade and antisaccade trials and covary with SRT, we extracted latencies for FEF and PEF activity for each participant. Virtual sensor latencies were determined by evaluating the time in which each participant's pseudo‐Z activity was 2*SD* above baseline. We also extracted peak pseudo‐Z values within the FEF at these latencies.

Four latencies per participant were extracted: *fast* antisaccade trials when the stimulus was on the (a) left and (b) right; *slow* antisaccade trials when the stimulus was on the (c) left and (d) right. A robust linear regression within R (rlm) using bisquare weighting in conjunction with the package *sandwich* and *jtools* for robust standard errors and *p*‐values was used to measure (a) temporal differences between prosaccade and antisaccade trials, (b) how well PEF and FEF latencies predicted antisaccade reaction time, and (c) how well FEF activity covaried with antisaccade reaction time. The model included main effects of SRT and the side the stimulus appeared (left or right), and the random effect was participants.

#### 2.5.2 Saccade‐execution (saccade‐aligned) analysis

To detect differences in neural activity prior to saccadic movement between prosaccade and antisaccade trials, contrast images were computed by subtracting source images between the two conditions (weights were calculated from combined prosaccade and antisaccade trials) using BrainWave. Combining prosaccade and antisaccade trials in the weights calculation ensures the images are not biased by differences in weights between the two tasks. The time at which there appeared to be a difference between prosaccade and antisaccade sources prior to movement was tested for significance using AFNI version 16.0.00 (Analysis of Functional NeuroImages). Event‐related images were normalized to the Talairach brain template and analyzed with a 2 (task: prosaccade vs. antisaccade) × 2 (stimulus side: left vs. right) repeated measures ANOVA in AFNI (Cox, 1996). To correct for multiple comparisons across the whole brain, resulting statistical maps were thresholded at a voxel‐wise level of *p* < .01 and a cluster size criterion of 12 voxels, resulting in a correct *p*‐value of *p* < .05. The cluster size criterion was determined by Monte Carlo simulations (10,000) conducted in AFNI program 3dClustSim using an FWHM of 9mm, as estimated by 3dFWHMx to estimate smoothness. This value was derived by computing a “null hypothesis” SAM image comparing the baseline period between the prosaccade and antisaccade conditions.

Given our hypothesis that neural activation within the contralateral FEF during antisaccade execution is related to the number of direction errors, we extracted virtual sensors, in pseudo‐Z, within contralateral FEF for each participant. Peak values were determined and the average pseudo‐Z values 10ms before and after the peak occurrence were evaluated. A robust binomial general linear model (glm) regression within *R* (glmrob) using the package *robustbase* in conjunction with the package *sandwich* and *jtools* for robust standard errors and *p*‐values was used to predict peak contralateral FEF power. For main effects, we used peak power and side of the visual stimulus and for random effects, we used intercepts for participants in the model.

The data that support the findings of this study are available on request from the corresponding author. The data are not publicly available due to privacy or ethical restrictions.

## 3 RESULTS

### 3.1 Behavioral results

**Figure 2:**
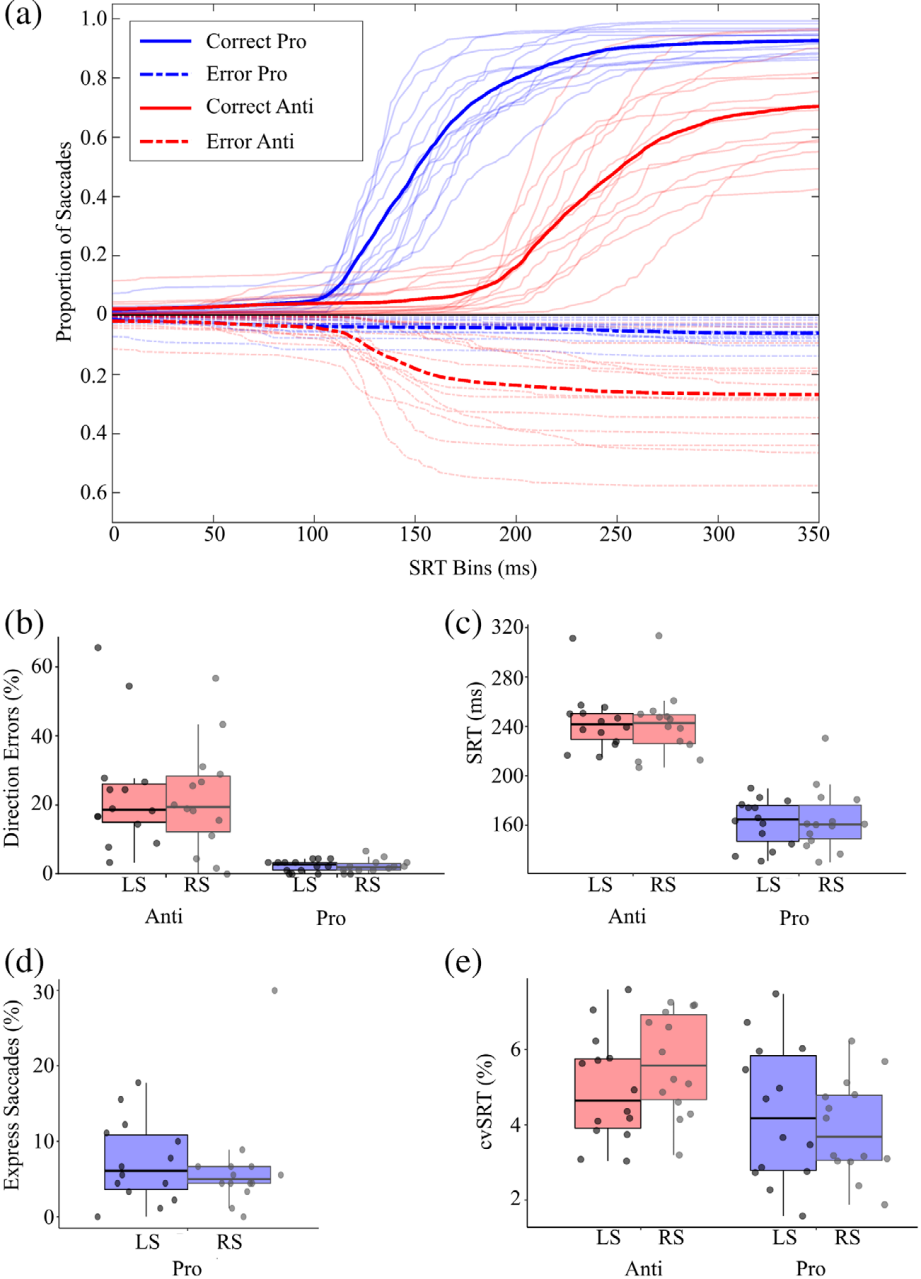
Eye movement behavioral data. (a) Cumulative histograms of SRT for prosaccade (light gray) and antisaccade (black) saccade distributions for all participants (thin lines) and the group average (thick lines). Positive Y values indicate correct saccades (solid lines), whereas negative Y vales indicate direction errors (dashed lines). (b) Mean percentage of direction errors (a saccade away from stimulus on prosaccade trial, toward stimulus on antisaccade trial) for stimuli on the left side (LS) and right side (RS). (c) Mean SRTs on correct trials for stimuli on the LS and RS. (d) Mean percentage of express saccades (90–135ms) for stimuli on the LS and RS. (e) Mean inter‐participant coefficient of variation in SRT (cvSRT) for stimuli on the LS and RS. SRT, saccade reaction time

Behavioral results for eye movements are shown in Figure 2. The cumulative distributions of SRTs for prosaccade and antisaccade trials are displayed for all participants (thin lines) and the group average (thick lines) (Figure 2a). Data are presented as a proportion of the total number of trials, where the latencies of the correct and incorrect saccades were categorized into SRT bins of 10ms increments. On average participants made 107 ± 30 (*SE*) out of 180 correct antisaccades and 137 ± 25 out of 180 correct prosaccades. We found no difference between the number of excluded trials in the left or right hemisphere for antisaccades (*t*(14) = −0.6, *p* = .57) and prosaccades (*t*(14)= 0, *p* = 1). A significant main effect of task was observed for SRT (*F*(1,14 = 160.20, *p* = 2e^−16^): anti‐SRTs were significantly longer than pro‐SRTs (Figure 2c). No effects were observed for stimulus side (*F*(1,14) = 0.00, *p* = .995) or stimulus side by task interaction (*F*(1,14) = 0.11, *p* = .72). A significant main effect for task was also observed for cvSRT (*F*(1,14) = 8.78, *p* = .0046): Anti‐SRTs were significantly more variable than pro‐SRTs (Figure 2e). There were no effects observed for stimulus side (*F*(1,14) = 0.150, *p* = .70) or stimulus side by task interaction (*F*(1,14) = 2.0, *p* = .16). The number of direction errors was modeled as a function of task, including saccade direction and participant as covariates. Regression analysis revealed that there were more direction errors on antisaccade trials than prosaccade trials (*b* = −0.226, SE =0.18, Z(14) = 9.86, *p* = 2e^−16^) (Figure 2b). We observed no differences in express saccades (SRT: 90–140ms) as a function of direction (*b* = −0.024, SE = 0.092, Z(14) = 0.78, *p* = .43) (Figure 2d).

### 3.2 Event‐related brain activity

#### 3.2.1 Saccade‐preparation (stimulus‐aligned)

Event‐related peak activity after fixation‐instructional cue appearance for left and right pro‐ and anti‐saccades was localized bilaterally in the primary visual cortex (BA 17; 150ms) and PEF (150ms), followed by bilateral activation of the FEF (240ms) (see Figure S2). No significant source amplitude differences were observed between task and direction after the fixation‐instructional cue. Differences in peak activation latencies were observed between pro‐ and anti‐saccade trials following the stimulus appearance (1,200ms after the fixation‐instructional cue; see Figure 1 for task timing information).

**Figure 3:**
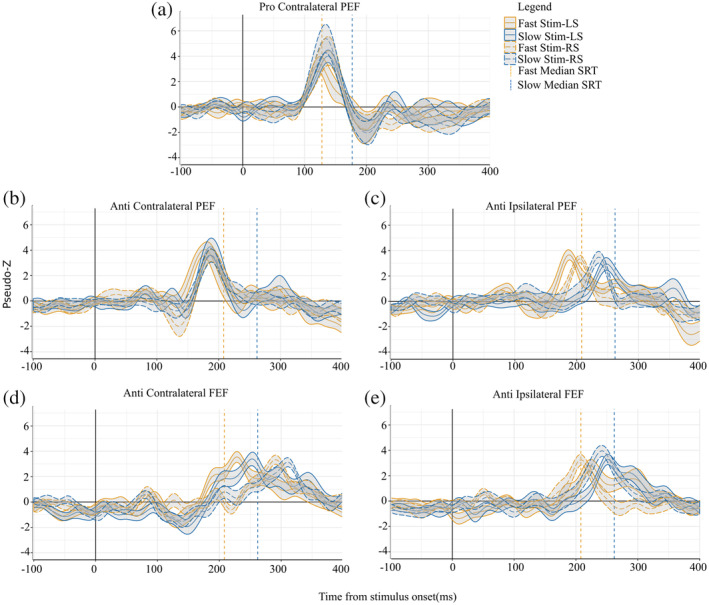
ERB Virtual‐Sensors (1–30Hz) from stimulus aligned trials (where 0 in the above plots are stimulus onset). All plots have both fast (orange) and slow (blue) virtual sensors for both left and right stimulus. Group median fast SRT is the orange vertical dashed line and group median slow SRT is the blue dashed vertical line for the plots corresponding task: either prosaccade or antisaccades. Shading around virtual sensor lines is standard error. (a) Peaks within contralateral PEF for prosaccade trials (fast and slow; stimulus on the right and left). (b) Peaks within contralateral PEF from antisaccade trials (fast and slow; stimulus on the right and left). (c) Peaks within ipsilateral PEF from antisaccade trials (fast and slow; stimulus on the right and left). (d) Peaks within ipsilateral FEF relative to the stimulus from antisaccade trials. (e) Peaks within contralateral FEF relative to the stimulus from antisaccade trials. ERB, event‐related beamforming; FEF, frontal eye field PEF; PEF, parietal eye field; SRT, saccade reaction time

Activity poststimulus appearance was analyzed after trials were separated into fast and slow SRTs based upon a median split of each participant's SRTs to reveal temporal dynamics within PEF and FEF for slow and fast saccades. For prosaccade trials, peak activations were observed in the PEF (BA 7) in the hemisphere contralateral to the visual stimulus shortly before the median pro‐SRT of fast trials (vertical black dashed line: 130 ± 15ms, Figure 3a). Peak activity for prosaccade trials within ipsilateral PEF occurred at 190ms and contralateral FEF (BA 6) at 240ms poststimulus, and this latency was slower than median SRTs (fast SRT = 130 ± 15ms and slow SRT = 180 ± 18ms). For antisaccade trials, contralateral PEF peaks occurred prior to median SRT at 190 ± 17ms (fast SRT = 213 ± 20ms and slow trials 263 ± 30ms) (Figure 3b). Mean ipsilateral PEF activity peaked at 240ms for fast trials and 280 ± 19ms for slow trials, both after the median SRT (Figure 3c). Figure S3 shows peak times across all participants within the PEF and across all antisaccade peaks, contralateral PEF and FEF, and ipsilateral PEF and FEF. We then compared differences between pro‐ and anti‐saccade peaks within the contralateral PEF since activity peaked prior to saccade movement. Regression analysis revealed that pro‐saccade PEF peak latencies were shorter than anti‐saccade PEF peak latencies (b = −0.28, 95%CI [−0.31, −0.25], *SE* = 0.014, Z(95) = −319.84, *p* = 2e^−16^).

The time course of activation within the ipsilateral and contralateral FEF for the anti‐saccade is shown in Figure 3 D and E, respectively. Ipsilateral FEF activation for both fast and slow trials peaked before their corresponding median SRTs. For fast trials, ipsilateral FEF peaked around 212 ± 25ms on the left and 210 ± 24ms on the right after stimulus appearance. Slow trials peaked slightly later at 250 ± 18ms for left stimulus trials and 240 ± 20ms for right stimulus trials. Even though peaks within contralateral FEF occurred after median SRT both for fast and slow trials, this activity started to increase above baseline before median SRT.

**Figure 4:**
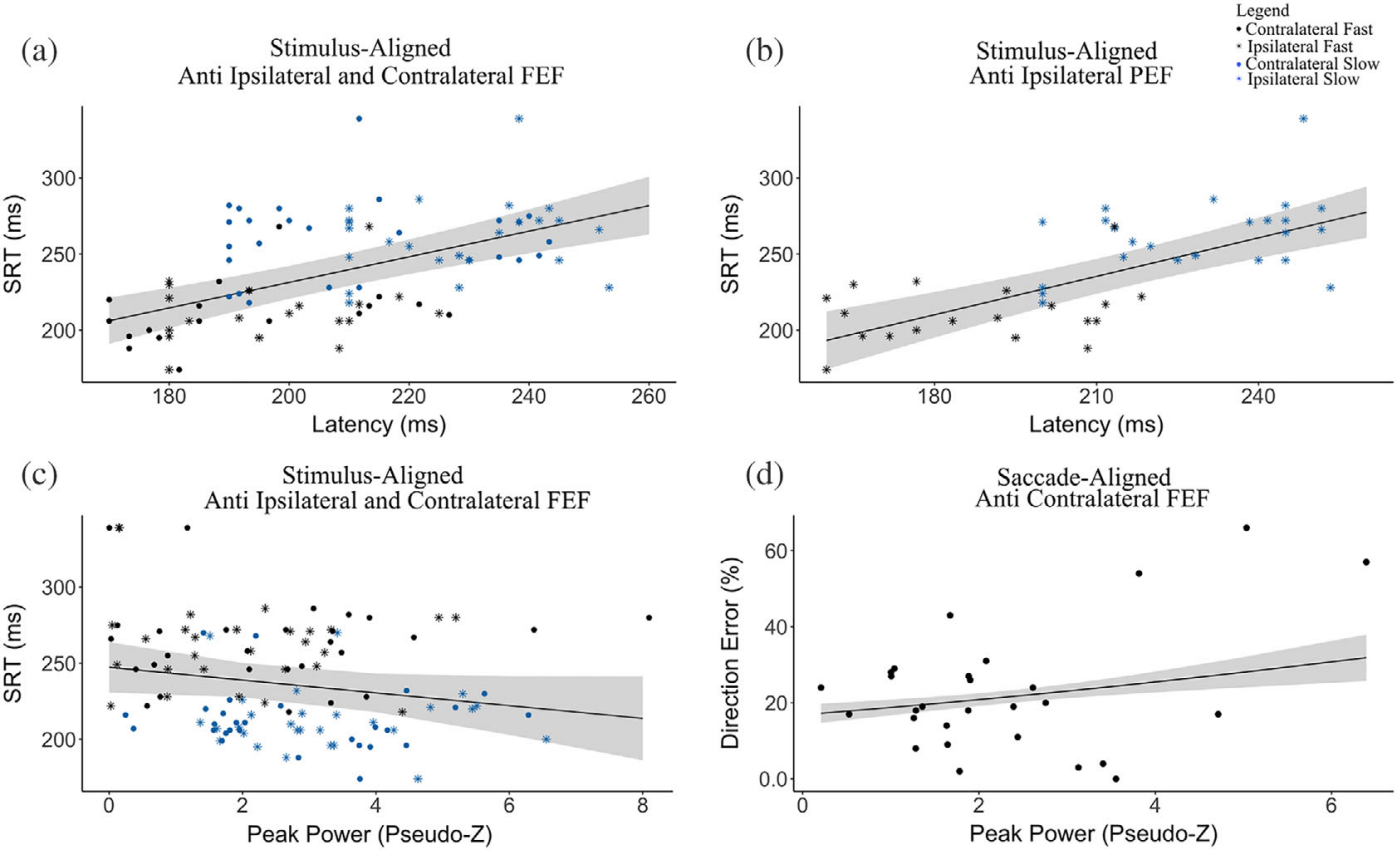
Plots from GLM fits between antisaccade measures (direction errors and SRT) and ERB measures (latency or mean peak pseudo‐Z) during antisaccade preparation (stimulus‐aligned) (a–c) and execution (saccade‐aligned) (d). (a) SRT and latency of peaks within ipsilateral and contralateral FEF (b) SRT and latency of peaks within ipsilateral PEF. (c) SRT and mean peak power pseudo‐Z within ipsilateral and contralateral FEF (d) Percent direction errors and mean peak power pseudo‐Z within contralateral FEF. GLM, general linear model; ERB, event‐related beamforming; FEF, frontal eye field PEF; PEF, parietal eye field; SRT, saccade reaction time

Given our hypothesis that the time course of activation of the FEF and PEF neural activation during saccade preparation is related to differences in SRT, we evaluated within‐participant SRTs and peaks for ipsilateral and contralateral FEF and latencies for ipsilateral PEF. Contralateral PEF was omitted because its mean peak latency occurred after median SRT. Median SRTs were modeled as a function of neural latency with covariates of stimulus direction and random effect of participant. This analysis revealed that delayed neural activity within both ipsilateral and contralateral FEF, as well as ipsilateral PEF, predicted longer SRTs (FEF, *R* ^2^ = 0.28, β = .84, 95%CI [0.58,1.15], *SE* = 0.14, *F*(3,95) = 33.16, *p* = 1.4e^−7^, Figure 4a; PEF, *R* ^2^ = 0.47, β = .87, 95%CI [0.58,1.15], SE = 0.15, *F*(2,47) = 35.14, *p* = 5.9 e^−7^, Figure 4b). We also modeled median SRTs as a function of pseudo‐Z with covariates of stimulus direction and random effect of participant. This analysis revealed that participants with longer SRTs have lower FEF activity: *R* ^2^ = 0.03, β = −4.84, 95%CI [−8.81,−0.86], *SE* = 2.41, *F*(2,47) = 4.12, *p* = .045 (Figure 4c ).

#### 3.2.2 Saccade‐execution (saccade‐aligned)

**Figure 5:**
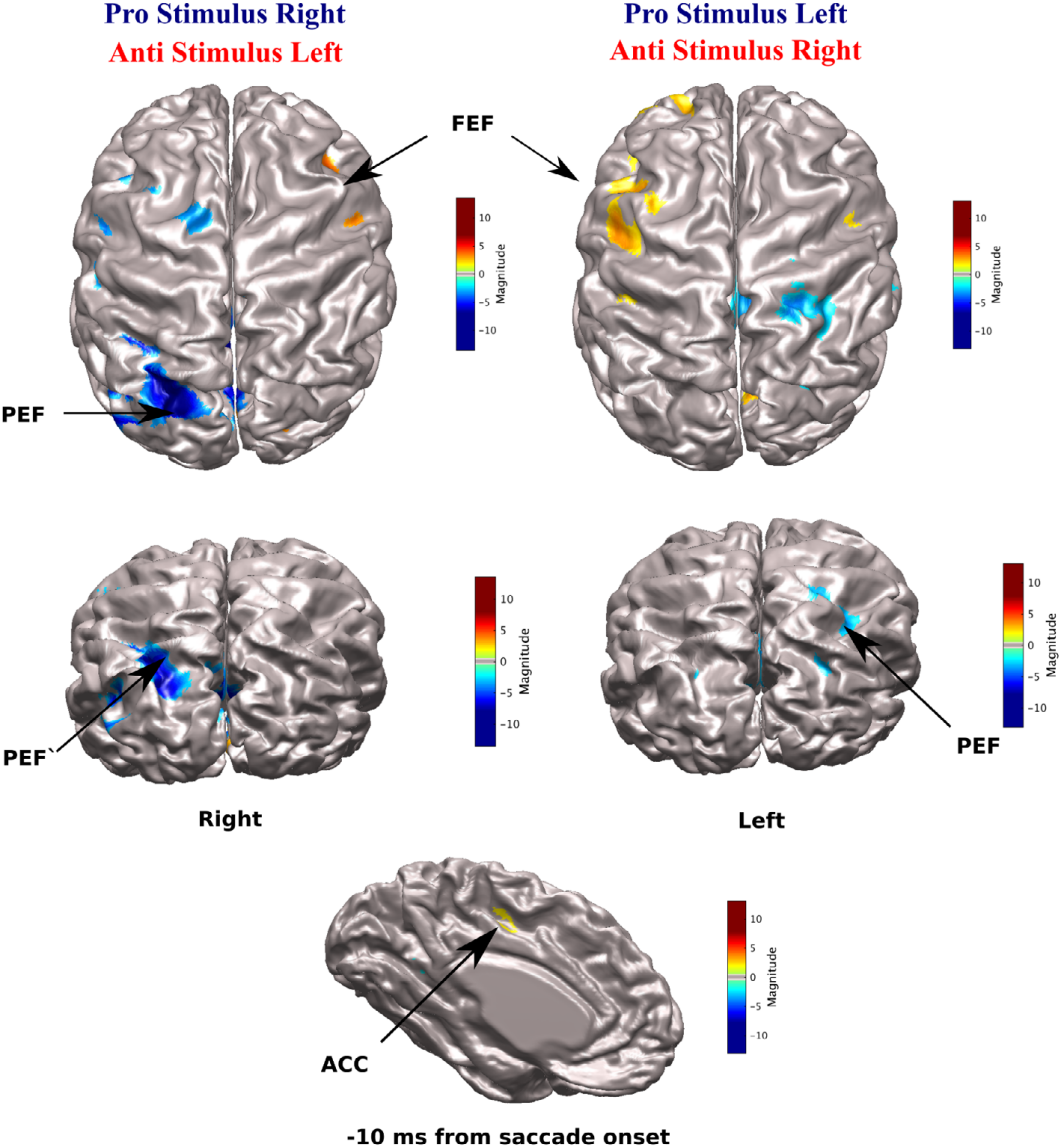
CIVET‐generated surface images with imposed ERB Beamforming contrast images of antisaccade (red) and prosaccade (blue) trials during saccade execution (saccade‐aligned). Virtual sensors were extracted FEF peaks (mean Talairach coordinates; left: *x* = −25, *y* = 2, z = 40; right: *x* = 26, *y* = 1, *z* = 40) and ACC peak (mean Talairach coordinates; *x* = 2, *y* = −4, *z* = 41) for antisaccade trials and PEF peaks (mean Talairach coordinates; left: *x* = −26, *y* = −57, *z* = 37; right: *x* = 20, *y* = −60, *z* = 35) in prosaccade trials at their corresponding peaks at 10ms before saccade movement. ERB, event‐related beamforming; FEF, frontal eye field PEF; PEF, parietal eye field; SRT, saccade reaction time

When we examined event‐related contrast images of prosaccade and antisaccade trials, aligned to the onset of saccade movement, differences between prosaccade and antisaccade tasks appeared 10ms prior to saccade‐onset (Figure 5 shows the source locations superimposed on the CIVET brain). In prosaccade trials, the PEF was activated contralateral to the stimulus (Figure 5, cool colors). For antisaccade trials, activity was observed in ipsilateral and contralateral FEFs and the posterior region of the anterior cingulate cortex (ACC) (Figure 5, hot colors). A 2 × 2 factor (task: prosaccade vs. antisaccade; stimulus side: left vs. right) repeated measures ANOVA was performed on source amplitude at 10ms before saccade movement. We observed a main effect of task within the left and right PEF (*F* = 4.32, *p* < .05, η^2^ = 0.83, 12 voxels) and FEF (*F* = −4.32, η^2^ = 0.38, *p* < .05, 15 voxels). Activation within the ACC (*p* > 0.05, voxles < 12) did not survive cluster correction.

**Figure 6:**
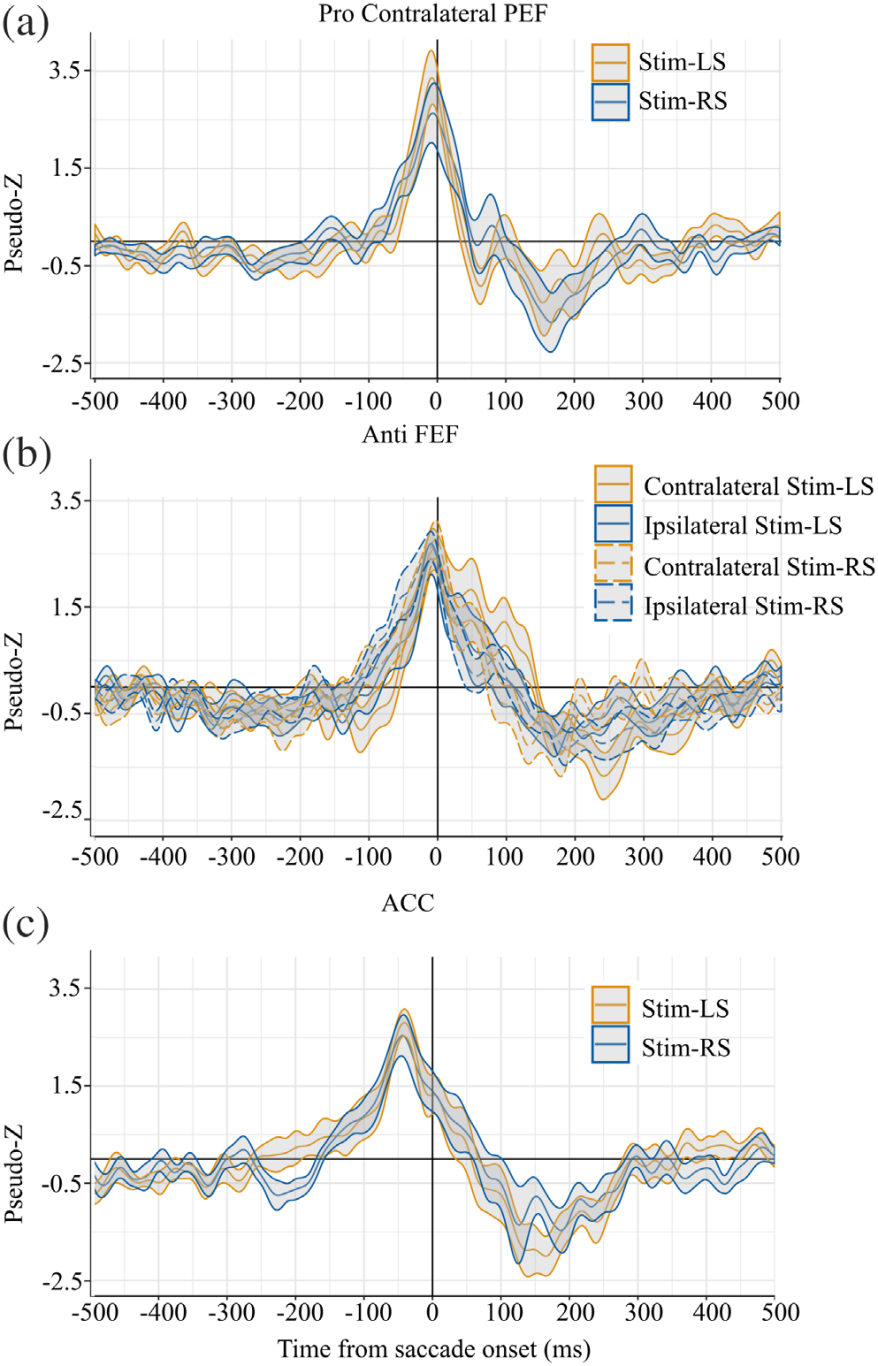
ERB Virtual‐Sensors (1–30Hz) from saccade‐aligned trials. Shading around virtual sensor lines is standard error. (a) Peaks within contralateral PEF were localized within prosaccade trails. (b) Peaks within contralateral and ipsilateral FEF were localized within antisaccade trials. (c) Peaks within the ACC were localized within antisaccade trials. ERB, event‐related beamforming; FEF, frontal eye field PEF; PEF, parietal eye field; SRT, saccade reaction time

Contralateral PEF peaks within prosaccade trials are shown in Figure 6a. For both left and right stimulus trials, PEF activity increased above baseline approximately 100ms prior to saccade onset, reached a maximum of 10ms before saccade onset and returned to baseline 100–200ms after movement. FEF activity for antisaccade trials is shown in Figure 6b. Contralateral and ipsilateral FEF activity began around 80ms prior to saccade onset and reached peak activity at approximately 10ms prior to saccade onset. Postsaccade activity within the ipsilateral FEF quickly decreased, while contralateral FEF activity had a longer return to baseline following movement onset.

We hypothesized that percent direction error from our behavioral analysis would be related to neural activation within contralateral FEF during saccade movement. We compared average pseudo‐Z values within contralateral FEF (−10ms to +10ms following each participant's contralateral FEF peak). Percent direction error was modeled as a function of pseudo‐Z with participant as a random effect. This analysis revealed that participants with more direction errors had increased power within the contralateral FEF, *R* ^2^ = 0.11, β = .24, 95%CI [0.18,0.31], *SE* = 0.12, Z(14) = 3.82, *p* = .00012 (Figure 4d). We also modeled median SRTs as a function of latency of peak evoked activity within FEF with participant being a random effect. No relation was found between peak FEF and SRT: *R* ^2^ = −0.01, β = −.20, 95%CI [−0.9,0.31], *SE* = 3.35, *Z*(14) = 2.59, *p* = .94.

## 4 DISCUSSION

Here, we describe the temporal dynamics of activation within human cortical areas associated with oculomotor control using MEG recordings combined with an interleaved prosaccade and anti‐saccade task and how they relate to SRT and direction errors. We report four novel findings based on our MEG analysis, which are organized around the stimulus‐ and saccade‐aligned results. From stimulus aligned data: (a) differences between prosaccade and antisaccade trials emerged within the PEF after stimulus appearance and before saccade onset; (b) on antisaccade trials, SRT increased along with the latency of neural activity after stimulus appearance within the ipsilateral PEF and bilateral FEF. Findings highlighted from saccade‐aligned data showed: (c) activity 10ms prior to saccade‐onset within ipsilateral and contralateral FEF was greater in antisaccade trials while activity with contralateral PEF was greater in prosaccade trials; and (e) increased activity within contralateral FEF within participants with high direction errors on antisaccade trials. Overall, we provide novel evidence for the role of ERB activity found in saccade preparation (stimulus‐aligned) and execution (saccade‐aligned) in prosaccade and antisaccade tasks. Implications of these findings are discussed below.

### 4.1 Saccade‐preparation (stimulus‐aligned)

We observed significant temporal differences within frontal and posterior cortical regions between prosaccade and antisaccade movement preparation. The PEF is involved in both sensory and motor processing and it projects to frontal oculomotor areas, such as the FEF (Ferraina, Paré, & Wurtz, 2002; Schall, 2015; Sestieri, 2008), SEF (Tobler & Müri, 2002), and DLPFC (Johnston & Everling, 2009), as well as the SCs (Paré & Wurtz, 1997). Previous structural imaging and lesion studies have demonstrated the importance of these areas in successful generation of voluntary antisaccades (Connolly et al., 2002; Guitton et al., 1985; Lee, Hämäläinen, Dyckman, Barton, & Manoach, 2011; Witiuk et al., 2014). The reason we did not observe event‐related activity (time‐ and phase‐locked) within the SEF nor DLPFC could be due to the fact these areas are more visible in induced oscillations as seen in decision‐making (Donner & Siegel, 2011; Womelsdorf & Fries, 2007). The SEF is important for internally guided decision making and sequencing of saccades (Coe, Tomihara, Matsuzawa, & Hikosaka, 2002) particularly for regulating the speed‐accuracy tradeoff (Stuphorn, Brown, & Schall, 2010). The DLPFC performs a modulatory function (Johnston & Everling, 2006a) by suppressing automatic or reflexive responses by sending inhibitory signals to oculomotor structures (Munoz & Everling, 2004; Pierrot‐Deseilligny, Rivaud, Gaymard, & Agid, 1991). Another important structure in competitive decision making of saccadic eye movement is the SCs (Coe et al., 2019), a deep brain structure, which results in low sensitivity in MEG beamforming due to its distance from the sensors and complex cytoarchitecture.

Preceding a prosaccade, we measured activation within the contralateral PEF 130ms after the stimulus, shortly before the mean SRT (163 ± 22ms). This timing was similar to a previous EEG study (130–150ms) (McDowell et al., 2005). We further observed peaks within ipsilateral PEF at ~190ms and within contralateral FEF at ~240ms poststimulus. Both of these peaks occurred after the mean SRT. However, other research groups that used a similar task without a gap period between fixation point disappearance and target/stimulus appearance, measured peaks within the PEF and FEF *prior* to saccade onset, which was approximately 260ms (McDowell et al., 2005; Sestieri, 2008). Here, we confirm with temporal specificity that prosaccades exhibit the short ocular‐motor circuit model of automatic saccades, which includes the PEF (Schiller & Tehovnik, 2005).

Neural activity postsaccade movement has no functional significance in eye‐movement control; however, a number of neurons exhibit directional and context‐dependent postsaccade activity in some eye‐movement tasks (Funahashi, Bruce, & Goldman‐Rakic, 1991; Genovesio, Brunamonti, Giusti, & Ferraina, 2007). These studies suggest that postsaccadic activities are related to cognitive behaviors, such as working memory (for a review see: Funahashi, 2001), attention (Yao, Treue, & Krishna, 2018), decision‐making (Teichert, Yu, & Ferrera, 2014) and performance monitoring (Stuphorn, Taylor, & Schall, 2000). For a postsaccadic activity review with different eye‐movement tasks, see the review by Funahashi (2014). Here, we will focus on results from the antisaccade task, where it has been shown that the inhibition signal is not needed for saccadic movement but is needed to improve performance (Coe et al., 2019). The inhibition signal is potentially sourced from the reshaping of automatic signals via voluntary signals in the frontal and parietal cortices or it may be a spatially focused inhibitory signal sent to the SCs from the basal ganglia (Amita, Kim, Smith, Gopal, & Hikosaka, 2019; Hikosaka et al., 2019; Watanabe & Munoz, 2011). Cortical postsaccade activity within the FEF has been proposed to be related to response‐evaluation to post‐decision outcomes and sensory information of the stimulus (Teichert et al., 2014). Hence, the FEF activity post prosaccadic movement seen in this study may be indicative of performance monitoring and optimizing subsequent behavior.

**Figure 7:**
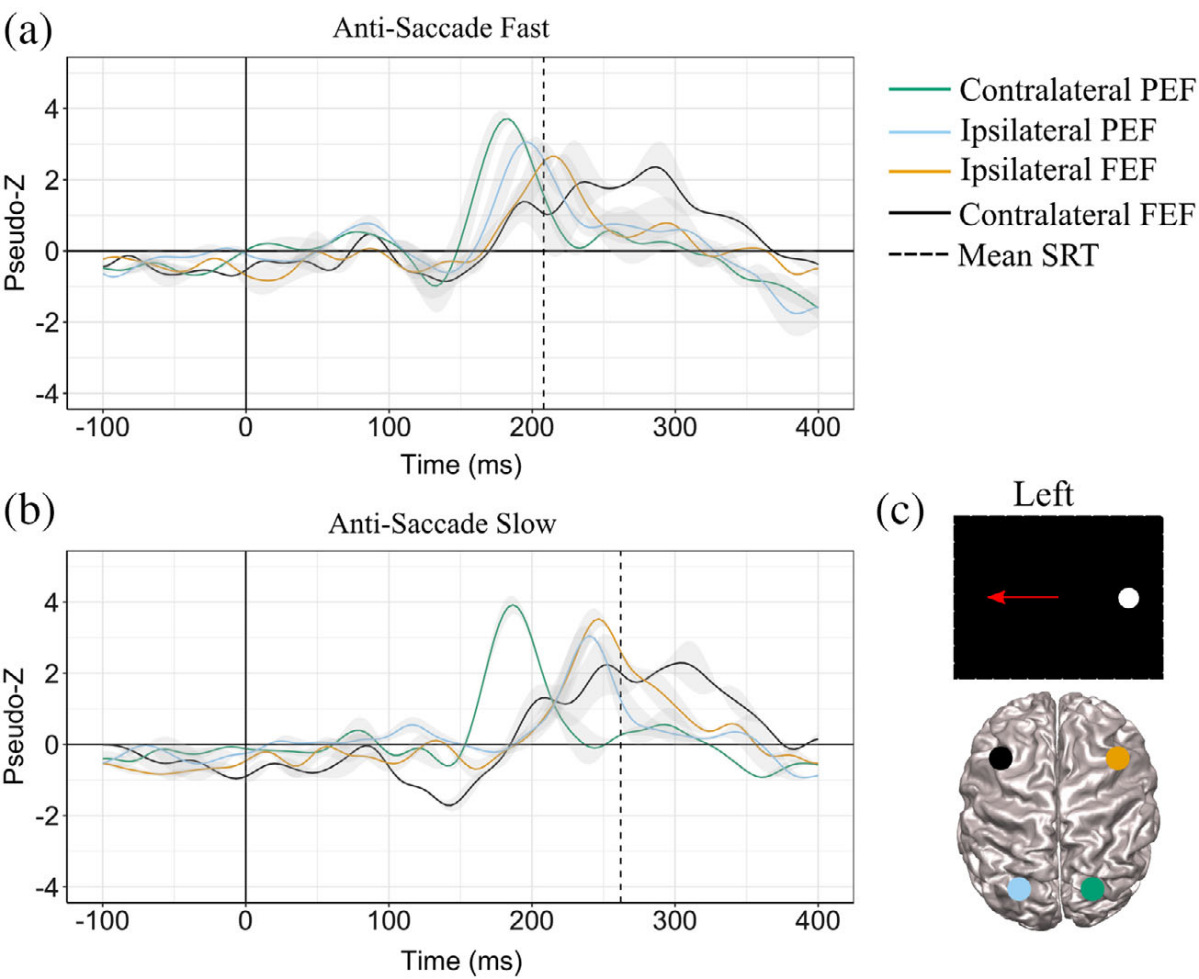
ERB Virtual‐Sensors (1–30Hz) from stimulus aligned antisaccade trials (where 0 in the above plots are stimulus onset). Plots show contralateral and ipsilateral PEF and FEF time courses for fast (a) and slow (b) trials averaged over left and right stimulus. Group median fast SRT is the black vertical dashed line in (a) and group median slow SRT is the black dashed vertical line in (b). (c) Is a representation of contralateral and ipsilateral PEF and FEF areas during left stimulus trial. ERB, event‐related beamforming; FEF, frontal eye field PEF; PEF, parietal eye field; SRT, saccade reaction time

Examination of presaccadic responses on correct antisaccade trials further permitted investigation of the role of the PEF and FEF regions in vector inversion. One may surmise that vector inversion initially occurs in PEF and then is mirrored within FEF. Our analysis of the timing of neural responses demonstrates a significant difference in timing from contralateral to ipsilateral PEF and then to ipsilateral FEF. A time course of activation averaged from left and right stimuli within PEF and FEF for fast and slow prosaccade and antisaccade trials are shown in Figure 7a,b, respectively (Figure 7c graphically depicts contralateral and ipsilateral PEF and FEF for the left stimulus). The temporal sequence relative to the stimulus of neural activity on correct anti‐saccade trials is as follows: (a) contralateral PEF ~185ms for both fast and slow trials, (b) ipsilateral PEF at 197ms for fast and 240ms for slow trials, and (c) followed by ipsilateral FEF at 220ms for fast and 245ms for slow trials. These findings are similar to prior EEG studies (Everling, Spantekow, et al., 1998; Moon et al., 2007), such that peaks within contralateral PEF (~160ms) were followed 30–90ms later by activity in the ipsilateral PEF. Monkey single‐unit recordings measured latencies within the LIP (the monkey homolog to the PEF) between 40 and 50ms (Bisley, Krishna, & Goldberg, 2004) and the FEF between 70 and 80ms (Schmolesky et al., 1998; Thompson et al., 1996), which is similar to the temporal sequence we observed here in healthy young adults. It is a challenge to directly compare invasive (single‐unit) recordings to MEG for a number of reasons: (a) the specific cellular and circuitry mechanisms that contribute to MEG signals is still unknown (Cohen, 2017); (b) macroscale signals from MEG suffer an ill‐posed inverse problem when attempting to deduce microscale properties. A recent study bridged the gap between invasive and noninvasive recordings in a different visual task (Sandhaeger, von Nicolai, Miller, & Siegel, 2019) and found that tuning of the two signals were similar in the extrastriata visual cortex (V4), but not in frontal areas (Sandhaeger et al., 2019), demonstrating that more work needs to be done to link invasive and noninvasive measures. Here, all the PEF and FEF peaks occurred prior to mean antisaccade movements for fast (213 ± 20ms) and slow (263 ± 30ms) trials. A limitation for most of the previous studies is that measurements were done on either PEF or FEF alone (monkey single‐unit recording (Bisley et al., 2004; Everling, Dorris, & Munoz, 1998; Schmolesky et al., 1998; Thompson et al., 1996). A small number of neuroimaging studies have measured vector inversion: one in EEG across posterior and frontal electrodes (Everling, Spantekow, et al., 1998), which has low spatial resolution, and another in MEG which measured sources within the left hemisphere only (Moon et al., 2007), likely due to low signal‐to‐noise ratios within their data set. We measured activation within PEF and FEF and demonstrated a neural activity pattern true to vector inversion. The presaccadic ipsilateral PEF activity observed within correct antisaccade trials suggests the role of this region in re‐mapping the visual response (Zhang & Barash, 2000; Zhang & Barash, 2004) in vector inversion, which is important for correct antisaccade movements. Furthermore, for a correct antisaccade movement, contralateral FEF must be inhibited prior to ipsilateral FEF movement (Bruce & Goldberg, 1985). Here, we demonstrate that activity within contralateral FEF peaked prior ipsilateral FEF; all of which suggests that vector inversion begins in PEF and is reflected in feedback‐generated activity in FEF (Figure 7).

Within antisaccade trials, higher FEF activity was associated with faster anti‐SRTs and this was in accordance with previous work (Dafoe, Armstrong, & Munoz, 2007; Fischer & Weber, 1992). Increased EEG negativity within frontal electrodes poststimulus presentation corresponded to shorter SRTs (Papadopoulou et al., 2010); fMRI studies (Connolly, Goodale, Goltz, & Munoz, 2005) found that FEF activity increased during the gap period (over 2 s) when SRT became shorter; and monkey neurophysiological studies (Everling & Munoz, 2000) found a relationship between higher contralateral FEF activity immediately before stimulus presentation and shorter SRT. Our results demonstrate the importance of prestimulus activity within the FEF on dictating SRT within voluntary saccades.

Here, we also demonstrated that shorter latency saccades were related to early activation within both the contralateral and ipsilateral FEF and ipsilateral PEF. Our findings are similar to previous work in MEG (Sestieri, 2008) which demonstrated that the FEF latency was faster with shorter SRT when divided into quartile bins. However, using a visually guided saccade task, this group found no significant difference in latency between PEF and FEF. No difference in latency between PEF and FEF implies different oculomotor mechanisms at play when producing a visually guided saccade versus an antisaccade. Within the antisaccade task, we demonstrated the latencies within all areas of the preparatory set—ipsilateral and contralateral FEF and ipsilateral PEF)—were related to individual SRT variability. The variability within the preparatory set, FEF and ipsilateral PEF, explained ~30% and ~50% of the behavioral variability, respectively. The explained variance between the preparatory set and behavior signifies how these cortical areas are involved in suppressive signals within the oculomotor system during antisaccades.

Overall, our results suggest that the preparatory set within the FEF and PEF plays a pivotal role in antisaccade planning and timing of execution needed for vector inversion. Furthermore, reaction time variability evolves from the build‐up of lags within motor preparation and inhibitory control stages.

### 4.2 Saccade‐execution (saccade‐aligned)

We observed differences between prosaccade and antisaccade MEG activity aligned to saccade‐onset within the FEF and PEF. Consistent with previous studies (Clementz, McDowell, & Stewart, 2001; Herdman & Ryan, 2007; McDowell et al., 2005; Moon et al., 2007; Papadopoulou et al., 2010; Richards, 2013), we observed that frontal brain activity was greater on antisaccade compared to prosaccade trials. During antisaccade trials, neurons tuned for triggering the automatic prosaccades must be inhibited prior to stimulus appearance to allow time for the voluntary antisaccade response to reach threshold, or a direction error occurs (Everling et al., 1999; Hanes & Schall, 1996). The structures involved in automatic saccade inhibition include the FEF (Everling & Munoz, 2000), SEF (Amador, Schlag‐Rey, & Schlag, 2004), and DLPFC (Johnston & Everling, 2009; Wegener et al., 2008). The ACC is involved in error detection (Johnston, Levin, Koval, & Everling, 2007), monitoring (Kerns et al., 2004; Polli et al., 2005), and antisaccade preparation (Ford, Goltz, Brown, & Everling, 2005). Notably, we also observed ACC activation prior to saccade initiation on antisaccade trials. However, this finding did not pass cluster thresholding—possibly due to the fact that the ACC is a deep brain structure making source detection more difficult with MEG, particularly with the relatively small number of trials used in the current study, unlike fMRI and single neuron recordings in monkeys which can more readily detect activity in deep brain structures. Another possibility is that activity within the ACC is not reflected in event‐related activity (time‐ and phase‐locked) and requires measurement of induced oscillations. Induced activity has been proposed to reflect global mechanisms such as top‐down attention and decision‐making (Donner & Siegel, 2011; Womelsdorf & Fries, 2007).

Interestingly, we observed that increased activity prior to saccade initiation within the contralateral FEF was associated with a greater number of directions errors. A similar relationship was observed in an fMRI study where increased activation within the FEF predicted antisaccade direction errors (Manoach et al., 2013). With the improved temporal resolution of MEG, we were able to confirm that this relationship is present prior to saccade initiation. Antisaccade direction errors reflect a breakdown in response inhibition, suggesting a failure of automatic saccade inhibition and voluntary saccade production. When a stimulus is presented, neural activity within the FEF contralateral to the stimulus increases, which is associated with movement towards the stimulus (i.e., prosaccades). Concurrently, activity within the ipsilateral FEF increases to initiate voluntary saccades (i.e., antisaccade). In monkeys, if inhibition is absent or weak within the ipsilateral FEF, an antisaccade direction error occurs because activity in the contralateral FEF surpasses the threshold prior to the ipsilateral FEF (Everling & Munoz, 2000). Here, using MEG, we indirectly demonstrated that this similar mechanism exists in humans.

Our results establish the temporal sequence of activity in cortical brain regions associated with saccade control in humans. Variation within our participants' activation or timing can be indirectly explained using lesion studies. Previous lesion studies demonstrate how damage to one region within the saccade network can affect saccade performance. Participants with higher direction errors could have abnormal activity within the PEF (Nyffeler, Rivaud‐Pechoux, Pierrot‐Deseilligny, Diallo, & Gaymard, 2007), where lesions result in impaired visual vector inversion or lesions within the DLPFC (Guitton et al., 1985; Pierrot‐Deseilligny et al., 1991). Longer antisaccade latencies may be related to abnormalities in the FEF due to its role in antisaccade initiation (Gaymard, Ploner, Rivaud‐Péchoux, & Pierrot‐Deseilligny, 1999; Rivaud, Müri, Gaymard, Vermersch, & Pierrot‐Deseilligny, 1994). To summarize, damage or injury to the PEF could affect vector inversion, while damage to frontal structures (FEF and DLPFC) could affect inhibitory control, leading to an increase in the number of direction errors. Consequently, differences in the number of direction errors and the timing of such errors affirm the distinct mechanisms of saccade suppression during the antisaccade task. Future studies are needed to further explore the variability in this mechanism between different populations, clinical or developmental, at the millisecond timescale using MEG.

The analysis approach used in the present study has some limitations. Here, we focused on time‐ and phase‐locked (aligned) activations using a signal‐averaged beamformer that is reflective of transient brain responses at the millisecond scale. Thus, we did not examine processes that are relatively slow (evolving over hundreds of milliseconds), which are reflective of interconnections within the brain, such as top‐down attention or inhibition (Donner & Siegel, 2011). Future directions include observing oscillatory responses related to pro‐ and anti‐saccades and the cross‐frequency relationships within the preparatory network. Furthermore, a caveat to this study is our use of a single‐source beamformer, which is known to reduce “highly” correlated source activity possibly affecting the precision of our bilateral PEF and FEF source waveforms. Multisource beamforming, such as multiple constrained minimum variance (MCMV), is applicable to bilateral sources that are highly correlated in phase and frequency (Herdman, Moiseev, & Ribary, 2018; Moiseev, Gaspar, Schneider, & Herdman, 2011; Moiseev & Herdman, 2013). Even with this advantage, MCMV has a few shortcomings: complicated source search and its dependence on signal to noise ratio (Herdman et al., 2018; Nunes et al., 2020). Based on this, future studies should include both single‐source and multisource beamformers to acquire a better estimate of the bilateral sources.

In the current study, we demonstrated that SRT varied with latencies measured from stimulus‐aligned activity within both ipsilateral and contralateral FEF and ipsilateral PEF on a millisecond timescale. We demonstrated that participants who had higher activity within the contralateral FEF produced more antisaccade direction errors—which may underlie a reduced ability to continuously suppress a prosaccade within antisaccade trials. Taken together, our findings motivate us to propose that cortical communication needed for oculomotor function is influenced by changes in evoked signal latency and activity, whereby the FEF and PEF are part of the cortical network that modulate changes in SRTs and direction errors.

## Supporting information


**Supplementary Figure 1** MEG channel data from MFL12 (frontal channel on the left; orange) along with ipsilateral FEF VS data (blue) for the same participant. Group median slow SRT is the black vertical dashed line; sensor‐level topography is centered at this time.
**Supplementary Figure 2**: CIVET‐generated surface images with imposed Event‐Related Beamforming images for left and right pro‐ and anti‐saccades of combined pro‐ and anti‐saccade trials relative to the instructional‐fixation‐cue appearance. At 150ms Visual cortex (BA 17) and parietal eye fields were localized and at 240ms bilateral frontal eye field were localized.
**Supplementary Figure 3**: Peak times across all participants represented as violin plots within A) PEF and across all anti‐saccade peaks B) contralateral PEF, ipsilateral PEF, contralateral FEF and ipsilateral FEF. Violin plots show the kernel probability density of the peak times for each area and included median and a box indicating the interquartile range.

## Data Availability

The data that support the findings of this study are available on request from the corresponding author. The data are not publicly available due to privacy or ethical restrictions.
