# Supplementary material for: Mapping neural dynamics underlying saccade preparation and execution and their relation to reaction time and direction errors
Source: Hum Brain Mapp. 2020 Jan 9;41(7):1934–49. doi: 10.1002/hbm.24922 (PMC7268073; doi:10.1002/hbm.24922)

Supplementary Figure 1: MEG channel data from MFL12 (frontal channel on the left; orange) along with ipsilateral FEF VS data (blue) for the same participant. Group median slow SRT is the black vertical dashed line; sensor-level topography is centered at this time.


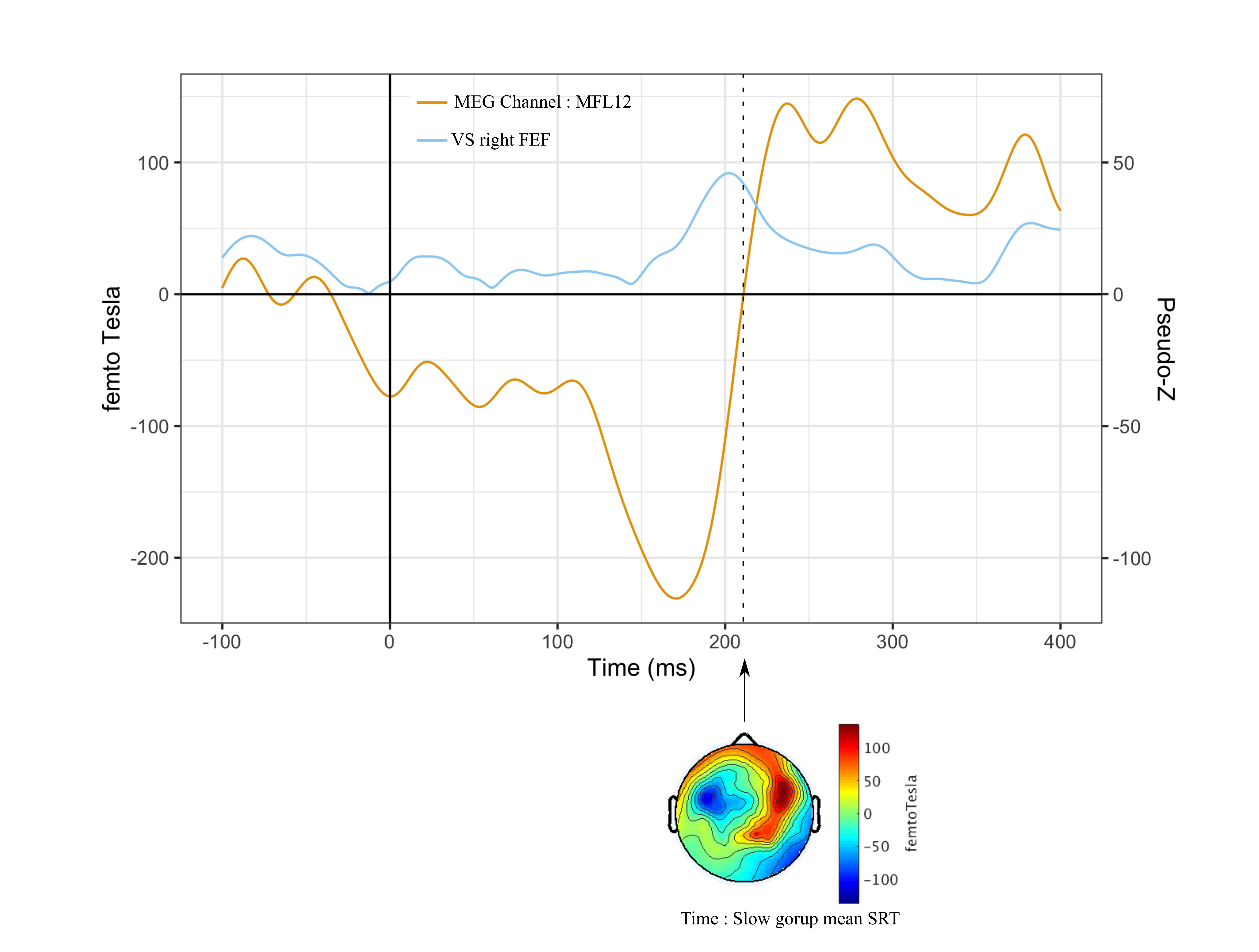


Supplementary Figure 2: CIVET-generated surface images with imposed Event-Related Beamforming images for left and right pro- and anti-saccades of combined pro- and anti-saccade trials relative to the instructional-fixation-cue appearance. At 150ms Visual cortex (BA 17) and parietal eye fields were localized and at 240ms bilateral frontal eye field were localized.


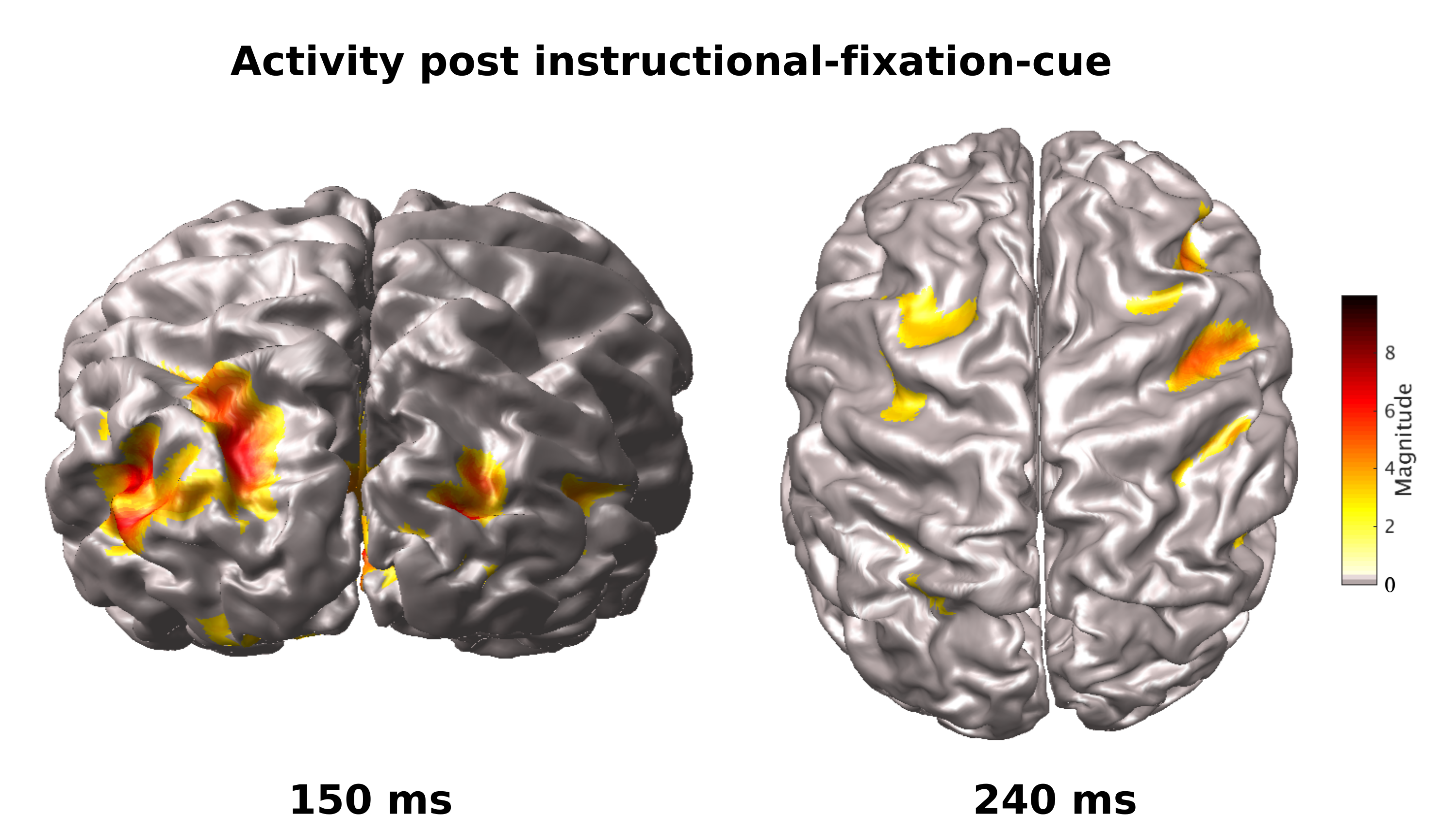


Supplementary Figure 3: Peak times across all participants represented as violin plots within A) PEF and across all anti-saccade peaks B) contralateral PEF, ipsilateral PEF, contralateral FEF and ipsilateral FEF. Violin plots show the kernel probability density of the peak times for each area and included median and a box indicating the interquartile range.


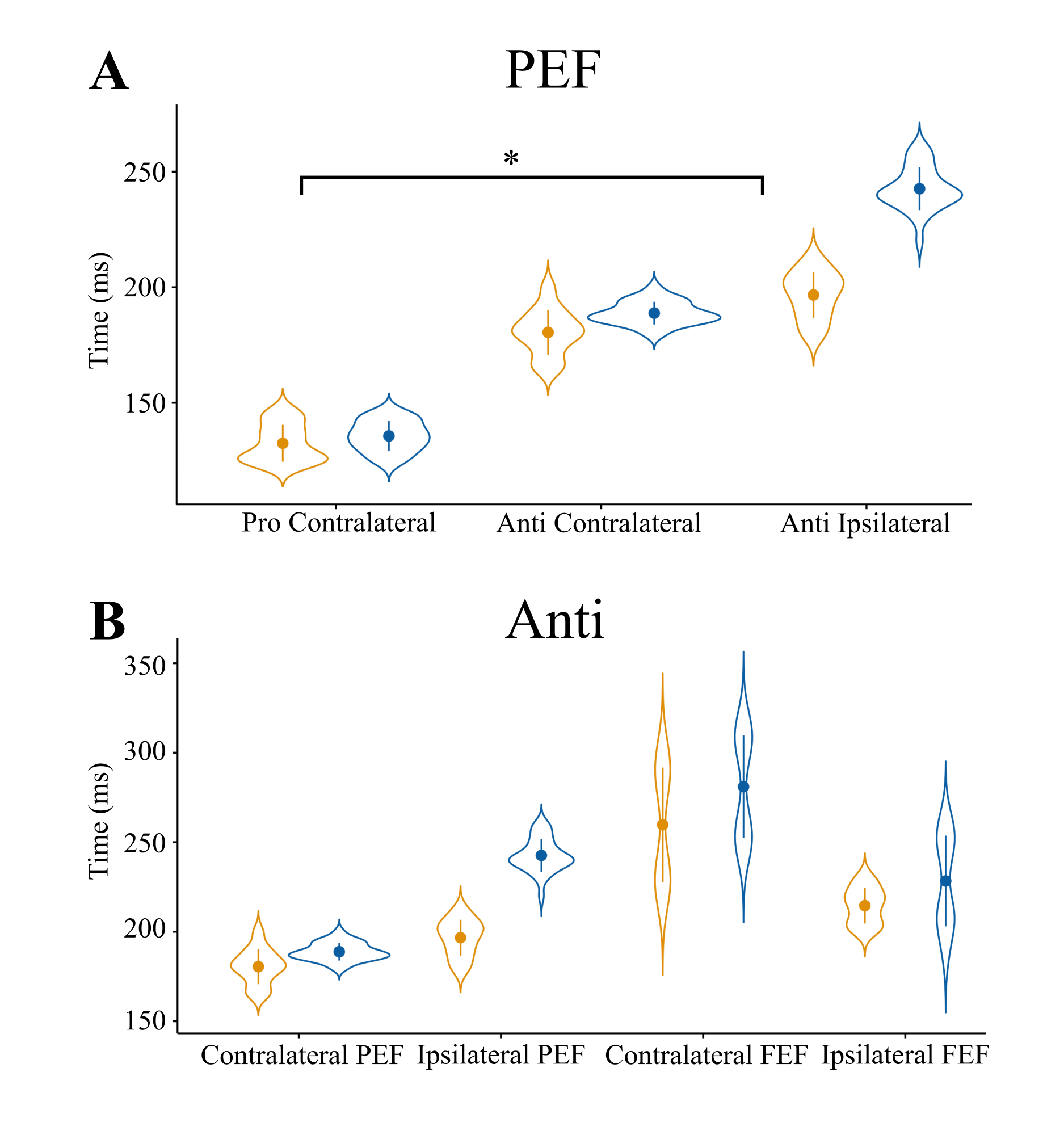

Supplement: Supplementary file 1 — Supplementary Figure 1 MEG channel data from MFL12 (frontal channel on the left; orange) along with ipsilateral FEF VS data (blue) for the same participant. Group median slow SRT is the black vertical dashed line; sensor‐level topography is centered at this time. Supplementary Figure 2: CIVET‐generated surface images with imposed Event‐Related Beamforming images for left and right pro‐ and anti‐saccades of combined pro‐ and anti‐saccade trials relative to the instructional‐fixation‐cue appearance. At 150ms Visual cortex (BA 17) and parietal eye fields were localized and at 240ms bilateral frontal eye field were localized. Supplementary Figure 3: Peak times across all participants represented as violin plots within A) PEF and across all anti‐saccade peaks B) contralateral PEF, ipsilateral PEF, contralateral FEF and ipsilateral FEF. Violin plots show the kernel probability density of the peak times for each area and included median and a box indicating the interquartile range. [file HBM-41-1934-s001.docx]
